# Ketamine reduces aversion in rodent pain models by suppressing hyperactivity of the anterior cingulate cortex

**DOI:** 10.1038/s41467-018-06295-x

**Published:** 2018-09-14

**Authors:** Haocheng Zhou, Qiaosheng Zhang, Erik Martinez, Jahrane Dale, Sile Hu, Eric Zhang, Kevin Liu, Dong Huang, Guang Yang, Zhe Chen, Jing Wang

**Affiliations:** 1 Department of Pain, The Third Xiangya Hospital and Institute of Pain Medicine, Central South University, Changsha, 410013 Hunan Province China; 2 Department of Anesthesiology, Perioperative Care and Pain Medicine, New York University School of Medicine, New York, 10016 NY USA; 3 Department of Psychiatry, New York University School of Medicine, New York, 10016 NY USA; 4 Department of Neuroscience and Physiology, New York University School of Medicine, New York, 10016 NY USA

## Abstract

Chronic pain is known to induce an amplified aversive reaction to peripheral nociceptive inputs. This enhanced affective response constitutes a key pathologic feature of chronic pain syndromes such as fibromyalgia. However, the neural mechanisms that underlie this important aspect of pain processing remain poorly understood, hindering the development of treatments. Here, we show that a single dose of ketamine can produce a persistent reduction in the aversive response to noxious stimuli in rodent chronic pain models, long after the termination of its anti-nociceptive effects. Furthermore, we demonstrated that this anti-aversive property is mediated by prolonged suppression of the hyperactivity of neurons in the anterior cingulate cortex (ACC), a brain region well known to regulate pain affect. Therefore, our results indicate that it is feasible to dissociate the affective from the sensory component of pain, and demonstrate the potential for low-dose ketamine to be an important therapy for chronic pain syndromes.

## Abstract (web summary)

Ketamine is a short-acting analgesic that also has anti-depressant effects. Here the authors show in rat models of chronic pain that low-dose ketamine can induce an anti-aversive state that persists after the initial short term analgesia has ended.

## Introduction

Pain has sensory and affective components, and a balanced interplay of the two components has evolved to form a vital protective function^1^. For example, tissue injury elicits anatomically specific nociceptive transmission, which in turn triggers aversion, a normal affective response that allows us to avoid further physical harm. In chronic pain conditions, however, patients demonstrate sensory hypersensitivity as well as increased affective or emotional responses. Previous research in chronic pain has primarily focused on abnormal sensory transmissions and suggests that the intense affective experience results from sensory hypersensitivity. However, in chronic pain syndromes such as fibromyalgia or persistent postoperative pain, patients develop magnified aversive responses, out of proportion to the intensity of peripheral nociceptive inputs in a wide-spread, anatomically nonspecific manner^2–6^. This generalized, anatomically nonspecific, enhancement of aversion leads to severe disability, and it suggests a pathological imbalance between the affective and sensory components in chronic pain conditions. Thus, mechanisms that specifically regulate the affective component of pain may have important therapeutic implications.

Studies in animal models indicate that altered synapses and circuits in the cerebral cortex contribute significantly to abnormalities in pain affect^7–10^. In particular, the anterior cingulate cortex (ACC) has been shown to play a key role in the aversive reactions to pain^11–21^. Furthermore, neurons in this region are known to undergo synaptic plasticity in the chronic pain state, resulting in enhanced output projections and an increased aversive response^7,8^. These results suggest that distinct cortical circuits have the potential to selectively regulate the affective valuation and response to noxious stimuli, and these cortical circuits may undergo maladaptive changes in the chronic pain state. However, so far, no particular pharmacological agent has emerged that can target these maladaptive cortical mechanisms to specifically control the affective symptoms of chronic pain.

Ketamine is a well-known analgesic with a half-life of 2–3 h. Since the anti-nociceptive property of ketamine is short-lived, its clinical use has been limited primarily to acute pain conditions. Despite its frequent use as a general anesthetic and short-acting analgesic, the effect of ketamine on the affective dimension of pain is not understood. Likewise, the analgesic targets for ketamine in the brain remain largely unknown. Recently, however, a single sub-anesthetic dose of ketamine has been shown to exert powerful antidepressant properties that last up to several days^22^. A likely mechanism for this enduring effect on mood and affect is the antagonism of excitatory *N*-methyl-d-aspartate (NMDA) receptors and subsequent restructuring of cortical circuits^23,24^. Thus, we hypothesized that ketamine could also alter cortical plasticity to selectively regulate the aversive response to peripheral nociceptive inputs in the chronic pain state.

We used a well-established conditioned place aversion test to assess the affective response to pain in rodents^8,19,25,26^. We found that chronic pain induced a generalized, anatomically nonspecific enhancement in pain aversion in a rat model. A single dose of ketamine, however, inhibited this abnormal enhancement in pain aversion. Furthermore, we were surprised to find that this anti-aversive effect lasted 5 days after a single administration. Finally, we demonstrated that these pain-inhibiting effects are mediated by a persistent suppression of hyperactivities in the ACC in the chronic pain state. Therefore, our results indicate that ketamine can be a novel therapy to selectively inhibit the affective symptoms of chronic pain.

## Results

### Chronic pain increases aversive responses to noxious stimuli

**Fig. 1:**
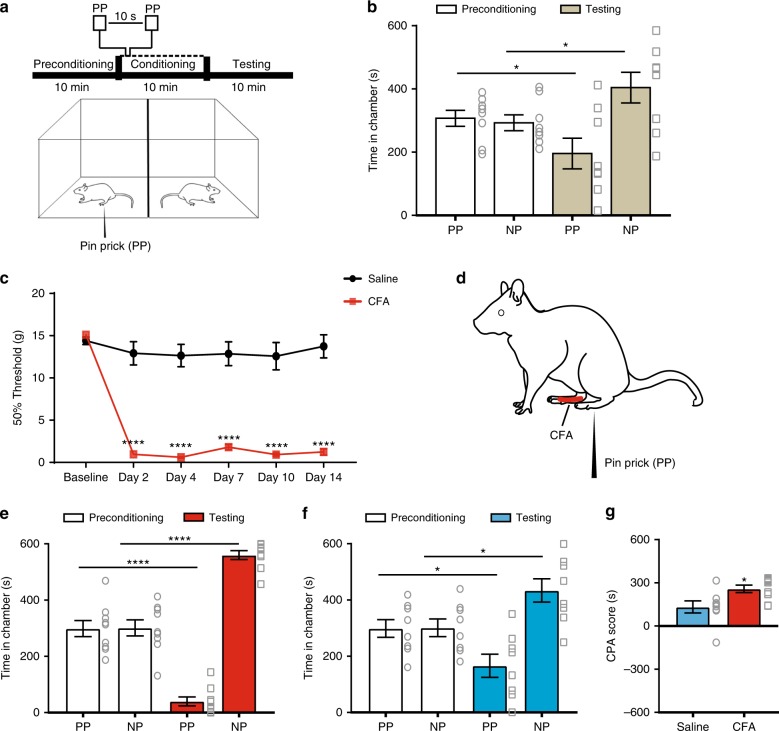
Chronic pain increases the aversive response to noxious peripheral stimulations. **a** We evaluated the aversive response to noxious stimulations in rats with a two-chamber conditioned place aversion (CPA) test. During the conditioning phase, one chamber was paired with pin prick (PP), while the other chamber was not paired with painful stimulation (NP). **b** Naive rats displayed avoidance of the chamber associated with acute pain (PP); *n* = 8; *p* = 0.0259, paired Student’s *t-*test. **c** CFA injection induced mechanical allodynia in the injured paw, *n* = 6; *p* < 0.0001. Two-way ANOVA with repeated measures and post-hoc Bonferroni test. **d** CPA assays were conducted by conditioning with PP in the uninjured paw contralateral to CFA injections. **e** CFA-treated rats demonstrated increased aversion to acute pain; *n* = 9; *p* < 0.0001, paired Student’s *t*-test. **f** Saline-injected rats showed normal pain aversion, *n* = 8; *p* = 0.0164. **g** Chronic pain induced enhanced aversive response, as demonstrated by increased CPA scores in CFA-treated rats, *n* = 8–9; *p* = 0.0194, unpaired Student’s *t*-test. Error bars represent S.E.M. ^∗^*p* < 0.05; ^∗∗∗∗^*p* < 0.0001

We used a two-chamber conditioned place aversion (CPA) assay to assess the aversive response to pain in rats^8,19,25,26^. During the preconditioning phase (10 min), rats were allowed free access to both chambers. During conditioning (10 min), one of the chambers was paired with repeated noxious mechanical stimulations (pin prick (PP)) of the hind paw, whereas the opposite chamber was not paired with noxious stimulations (NP). Finally, during the test phase (10 min), rats were allowed free access to both chambers again without stimulations (Fig. 1a). The aversive response to pain was quantified by a CPA score, which was calculated by subtracting the time rats stayed in the PP chamber during the test phase from the preconditioning phase^8,27^.

As expected, rats developed avoidance of the chamber associated with PP stimulation, indicating normal aversive responses to noxious stimuli (Fig. 1b). To study the aversive processing in the chronic pain condition, we injected complete Freund’s adjuvant (CFA) into the hind paws of rats to induce inflammatory pain. CFA injections induced sensory hypersensitivity, manifested by mechanical allodynia (Fig. 1c). To specifically investigate the impact of chronic pain on the aversive response to noxious stimulations, during the conditioning phase of the CPA, we paired one chamber with PP stimulation of the uninjured paws (contralateral to sites of CFA injections) (Fig. 1d). In this way, the CPA results would not be confounded by sensory hypersensitivity in the injured paws, and rather represented aversive reactions to noxious inputs in general. We found that rats that received CFA injections, compared with control rats that received saline injections, showed an increased aversive response to noxious stimulations of the uninjured paws (Fig. 1e, f). This difference can be quantitated by an elevated CPA score in CFA-treated rats (Fig. 1g). To ensure that this CPA protocol is robust enough for the testing of the aversive response to acute pain, we increased the conditioning period to 30 min (Supplementary Fig. 1). We found no difference in the aversive scores between 10 and 30 min of conditioning.

These data indicate that rats with chronic pain develop an enhanced aversive response to peripheral noxious inputs in a generalized, anatomically nonspecific manner. These results are in agreement with previous preclinical studies on the aversive response to acute thermal stimulation in the rodent chronic pain model^8^. They also mirror findings of increased aversive reactions to low-grade peripheral noxious inputs in a diffuse anatomical distribution in chronic pain syndromes such as fibromyalgia^2–6^.

### Ketamine provides persistent relief of pain aversion

**Fig. 2:**
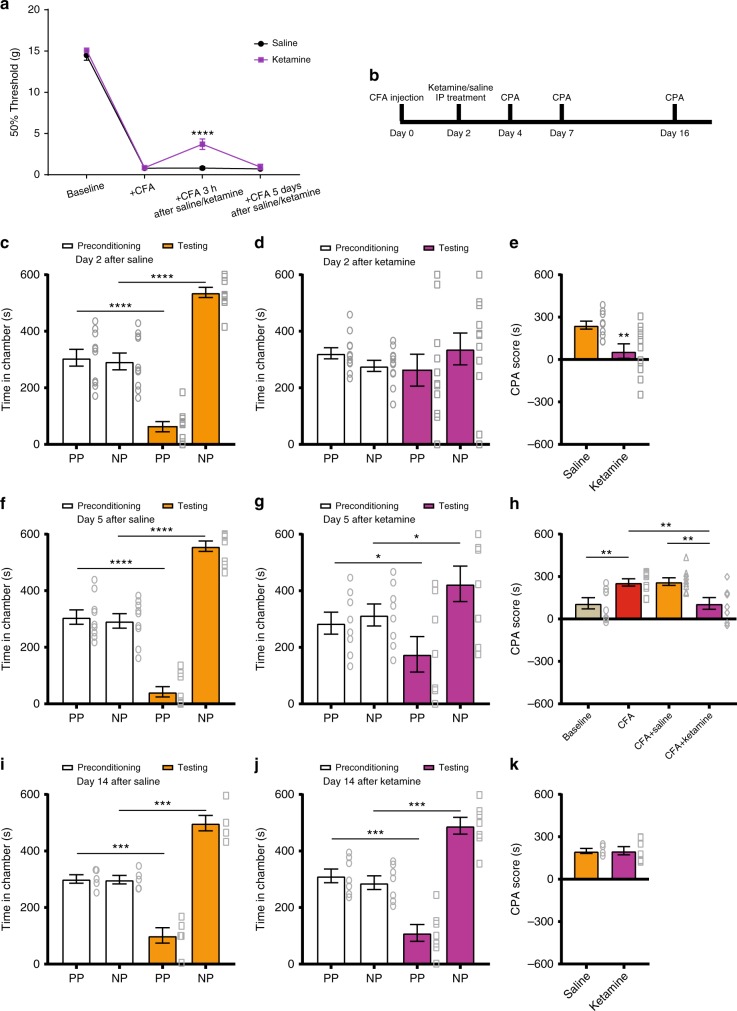
Ketamine provides long-lasting inhibition of affective symptoms of chronic pain. **a** A single sub-anesthetic dose of ketamine (10 mg kg^−1^) provides only transient relief of allodynia; *n* = 9–10; *p* < 0.0001. **b** Time course for CPA tests in ketamine-treated rats. **c**–**e** A single dose of ketamine inhibited the aversive response to acute pain in CFA-treated rats for at least 2 days. **c** CPA results 2 days after IP saline administration; *n* = 10; *p* < 0.0001, paired Student’s *t*-test. **d** CPA results 2 days after ketamine administration; *n* = 11; *p* = 0.2731. **e** CFA-treated rats demonstrate lower CPA scores 2 days after ketamine administration; *n* = 10–11; *p* = 0.0067, unpaired Student’s *t*-test. **f**–**h** A single dose of ketamine reduced the aversive response to acute pain in CFA-treated rats up to 5 days. **f** CPA results 5 days after saline administration; *n* = 9; *p* < 0.0001, paired Student’s *t*-test. **g** CPA results 5 days after ketamine administration; *n* = 8; *p* = 0.0333. **h** Ketamine restored the aversive response in chronic pain rats to baseline (−CFA) levels; *n* = 8–9; *p* = 0.0063, unpaired Student’s t-test. **i**–**k** The anti-aversive effect of ketamine in CFA-treated rats was eliminated 14 days after administration. **i** CPA results 14 days after saline administration; *n* = 5; *p* = 0.0003, paired Student’s *t*-test. **j** CPA results 14 days after ketamine administration; *n* = 7; *p* = 0.0005. **k** CPA scores for ketamine was similar to saline 14 days after administration; *n* = 5–7; *p* = 0.9665, unpaired Student’s *t*-test. Error bars represent S.E.M. ^∗^*p* < 0.05; ^∗∗^*p* < 0.01; ^∗∗∗^*p* < 0.001; ^∗∗∗∗^*p* < 0.0001

We tested if ketamine could reduce this pathologically elevated aversive response in the chronic pain condition. We chose a sub-anesthetic dose of ketamine (10 mg kg^−1^) based on previous studies of depression^23,28,29^. The half-life of the anti-nociceptive effects of ketamine is known to be approximately 3 h. Thus, as expected, ketamine administration caused only transient relief of sensory allodynia (Fig. 2a). We then performed CPA assays to assess the aversive component of pain after ketamine or saline administration (Fig. 2b). In order to specifically test the impact of ketamine on the aversive symptoms of chronic pain, we waited for the resolution of its anti-nociceptive effects to perform the CPA tests. We found that 2 days after a single dose of ketamine, CFA-treated rats demonstrated a dramatic reduction in their aversive response to pain, compared with control rats that received saline injections (Fig. 2c, d). This reduction was validated by significantly decreased CPA scores in the ketamine group (Fig. 2e).

Previous psychiatric studies have shown that ketamine can produce antidepressant effects that last multiple days after a single administration. Thus, we performed CPA tests 5 days after a single injection of ketamine or saline. We found that CFA-treated rats continued to demonstrate a decrease in their aversive response to acute pain inputs 5 days after a single administration of ketamine (Fig. 2f, g). In fact, comparisons with rats that did not experience chronic pain demonstrated that a single dose of ketamine restored the aversive response of CFA-treated rats to baseline levels for at least 5 days (Fig. 2h). This prolonged relief of pain aversion contrasts sharply with the short duration of the anti-nociceptive effects of ketamine (Fig. 2a). This ability to produce enduring relief of the abnormal aversive experience, long after the termination of its effect on sensory hypersensitivity, suggests that ketamine can successfully dissociate the affective from the sensory component of chronic pain. By providing this persistent dissociation of the affective from the sensory component of pain, ketamine may be particularly useful in treating diseases such as fibromyalgia, where patients report chronic magnified emotional responses to wide-spread nociceptive inputs^2,3^. Finally, on day 14 after a single administration of ketamine, we did not observe any anti-aversive effects (Fig. 2i–k).

**Fig. 3:**
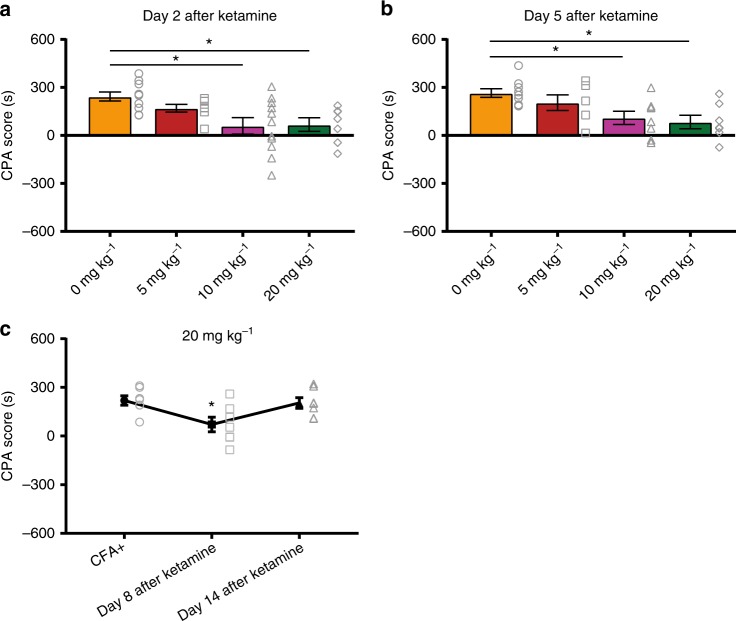
A dose–response study of the anti-aversive effects of ketamine. **a** CPA results 2 days after ketamine treatment. The anti-aversive effect of ketamine was observed at the doses of 10 mg kg^−1^, *n* = 11, *p* = 0.0101, and 20 mg kg^−1^, *n* = 7, *p* = 0.0392, but not 5 mg kg^−1^; *n* = 7, *p* > 0.9999. One-way ANOVA and post-hoc Bonferroni test. **b** CPA data 5 days after ketamine treatment. The anti-aversive effect of ketamine lasted at least 5 days at doses of 10 mg kg^−1^, *n* = 8, *p* = 0.0388, and 20 mg kg^−1^, *n* = 7, *p* = 0.0153, but not 5 mg kg^−1^; *n* = 6, *p* > 0.9999. **c** Enduring anti-aversive effects were observed at the 20 mg kg^−1^ dose. CPA scores were reduced for at least 8 days after IP administration of ketamine; *n* = 7; *p* = 0.0405. One-way ANOVA with repeated measures and post-hoc Bonferroni test. Error bars represent S.E.M. ^∗^*p* < 0.05

Next, we constructed a dose–response study for ketamine. We administered 5 and 20 mg kg^−1^ doses, in addition to 10 mg kg^−1^, to CFA-treated rats (Fig. 3). When we compared the CPA scores, we found that 5 mg kg^−1^ did not produce any significant anti-aversive effects, even 2 days after its administration (Fig. 3a). In contrast, both 10 and 20 mg kg^−1^ provided significant anti-aversive effects in rats with chronic pain at multiple days after administration (Fig. 3a, b). In the case of 20 mg kg^−1^, this effect lasted at least 8 days (Fig. 3c). By day 14, however, we could not observe any anti-aversive effects of ketamine. These results suggest that a single dose of ketamine can produce between 8 and 14 days of relief for the aversive experience of chronic pain.

**Fig. 4:**
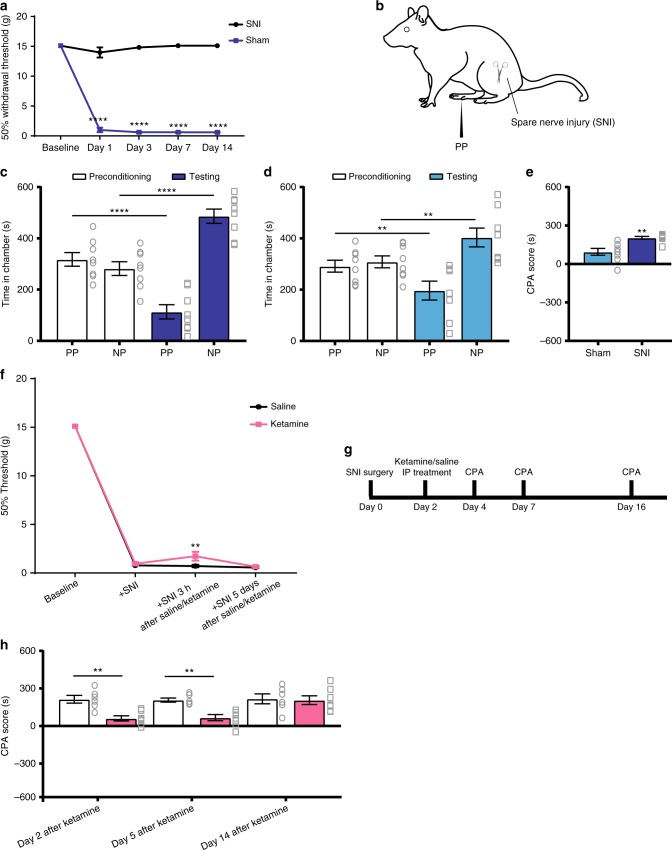
Ketamine reduced the enhancement in pain aversion in a chronic neuropathic pain model. **a** Rats developed persistent allodynia after SNI surgeries, *n* = 6; *p* < 0.0001. Two-way ANOVA with repeated measures and post-hoc Bonferroni test. **b** Schematic of the CPA testing of the SNI-treated rats. **c** The aversive response to acute noxious stimulations was enhanced in SNI-treated rats; *n* = 8; *p* < 0.0001, paired Student’s *t*-test. **d** Rats displayed normal level of avoidance of the chamber associated with PP after sham surgery; *n* = 8; *p* = 0.0091. **e** SNI induced generalized enhancement of aversion to noxious stimulations, as demonstrated by the increased CPA score; *n* = 8; *p* = 0.0019, unpaired Student’s *t*-test. **f** A single sub-anesthetic dose of ketamine (10 mg kg^−1^) provided transient relief of mechanical allodynia in SNI-treated rats; *n* = 6–8; *p* < 0.0084. Two-way ANOVA with repeated measures and post-hoc Bonferroni test. **g** Timeline for the CPA experiment in SNI-treated rats. **h** The anti-aversive effect of ketamine on SNI-treated rats was present 2 days (*n* = 6–7; *p* = 0.0021) and 5 days (*p* = 0.0048) but not 14 days (*p* > 0.9999) after its administration, two-way ANOVA with repeated measures and post-hoc Bonferroni tests. Error bars represent S.E.M. ^∗∗^*p* < 0.01; ^∗∗∗∗^*p* < 0.0001

To confirm these striking anti-aversive effects of ketamine in the chronic pain state, we tested a second chronic pain model in rats—a spared nerve injury (SNI) model. In contrast to the CFA model, which induces persistent inflammatory pain, the SNI model mimics chronic neuropathic pain as the result of peripheral nerve injury (Fig. 4a, b). Similar to what we found with the CFA model, the aversive response to acute noxious stimulations in the opposite (uninjured) paw was significantly enhanced in SNI-treated rats, compared with rats that underwent sham surgery (Fig. 4c–e). These results confirm the finding that chronic pain induces an anatomically nonspecific increase in aversion^8,30^. When we injected ketamine into these rats, we did not observe long-lasting anti-nociceptive effects, as manifested by only a transient relief of mechanical allodynia (Fig. 4f). In contrast, when we tested the effects of ketamine on the aversive response to pain (Fig. 4g), we found that the CPA scores were reduced for at least 5 days (but not more than 14 days) after a single administration (Fig. 4h). These results are compatible with what we found in the CFA model, and indicate that a single administration of ketamine can produce anti-aversive effects for several days in multiple chronic pain conditions.

### Ketamine reduces hyperactivity of ACC neurons

To understand the mechanism for this persistent inhibition of pain aversion, we studied the effect of ketamine on the ACC. ACC neurons are known to alter their firing rates^31–35^ to regulate the affective symptoms of pain^13,19,20,36^. After chronic pain, however, neurons in the ACC have been shown to undergo synaptic plasticity^7,8^. Increased activities in these neurons, in turn, have been hypothesized to produce enhanced aversive responses in chronic pain conditions^8,18,19,37,38^.

**Fig. 5:**
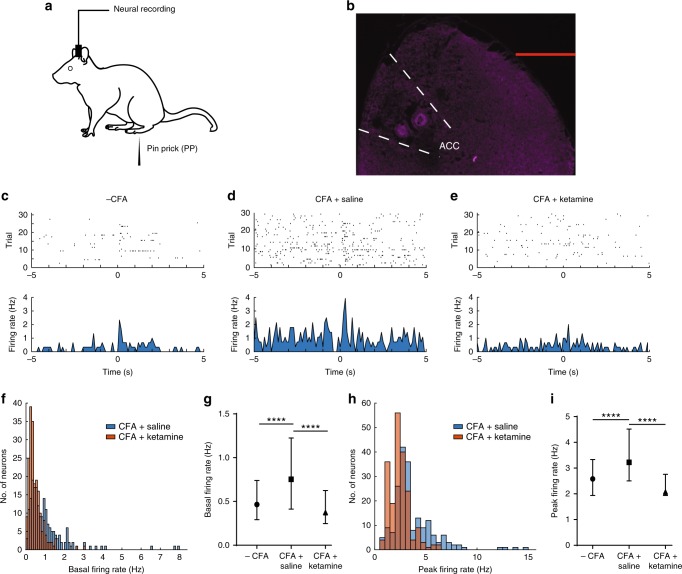
Ketamine reduces hyperactivity of neurons in the ACC. **a** Experimental paradigm for electrophysiological recordings in free-moving rats. **b** Location of recording electrodes in the ACC. **c** Raster plots and peri-stimulus time histograms (PSTHs) of representative ACC neurons. Time 0 indicates the onset of noxious (PP) stimulation. **d**, **e** Raster plots and PSTHs for CFA-treated rats 5 days after receiving **d** saline or **e** ketamine infusion. **f**, **g** Chronic pain increased basal firing rates of ACC neurons, but ketamine treatment inhibited this increase. **f** Histogram showing the distribution of basal firing rates of neurons after saline or ketamine treatment in CFA-treated rats. **g** Median ± interquartile range for basal firing rates in naive rats, and in CFA-treated rats after ketamine or saline injection; *n* = 201 (−CFA), 195 (+CFA and saline), and 200 (+CFA and ketamine); *p* < 0.0001, Kruskal–Wallis test with post-hoc Dunn’s multiple comparisons. See Methods. **h**, **i** Ketamine inhibited the enhancement of pain-evoked firing rates of ACC neurons in CFA-treated rats; *p* < 0.0001. **h** Histogram showing the distribution of peak firing rates for neurons after saline or ketamine treatment in CFA-treated rats. **i** Median ± interquartile range for pain-evoked firing rates in naive rats, and in CFA-treated rats after ketamine or saline injection; *n* = 201 (−CFA), 195 (+CFA and saline), and 200 (+CFA and ketamine); *p* < 0.0001. Error bars represent S.E.M. Scale bar equals 1000 μm in **b**. ^∗∗∗∗^*p* < 0.0001

We performed extracellular recordings in the ACC of awake, free-moving rats (Fig. 5a, b). Compatible with previous findings^8,31–35^, PP increased the firing rates of individual neurons (Fig. 5c). We recorded from layers 5–6 in the ACC and, as expected^39–41^, a majority of the cortical neurons we recorded were pyramidal neurons (Supplementary Fig. 2). We then measured the response of ACC neurons to acute pain in the chronic pain state. We injected CFA into paws ipsilateral to the ACC recording sites, and 2 days later, we administered either ketamine or saline (control) into the rats. At 5 days after ketamine/saline administrations, we recorded ACC activities with PP stimulation of the contralateral, uninjured paws (Fig. 5d, e). To quantify the effect of ketamine on individual ACC neurons, we calculated basal and pain-evoked firing rates. Rats in chronic pain showed increases in both basal (Fig. 5f, g) and evoked firing rates (Fig. 5h, i), compared with baseline, pre-CFA conditions. Elevated basal firing indicates hyperexcitability of these neurons at rest, consistent with results from previous in vitro studies^7,42^. On the other hand, an increase in stimulus-evoked firing rates demonstrates that, in the chronic pain state, individual ACC neurons amplify their response to peripheral nociceptive inputs. This hyperactivity of the ACC has been shown in previous studies to contribute to enhanced affective responses in the chronic pain state^7,8^. Ketamine, however, dramatically reduced both basal (Fig. 5f, g) and stimulus-evoked firing rates (Fig. 5h, i) of individual ACC neurons in CFA-treated rats. In fact, ketamine treatment returned both basal and pain-evoked firing rates of these neurons in CFA-treated rats to their baseline, pre-CFA levels, and this inhibitory effect on ACC hyperactivity lasted at least 5 days (Fig. 5g, i). These results indicate that a single dose of ketamine provides persistent suppression of the hyperactivity of individual ACC neurons in the chronic pain state.

### ACC inhibition mediates ketamine’s anti-aversive effects

**Fig. 6:**
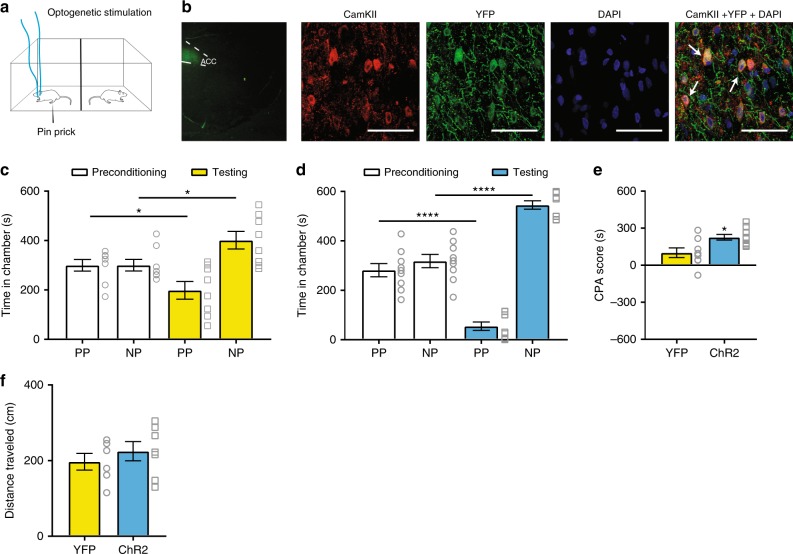
Ketamine inhibits ACC activities to reduce pain aversion. **a** Schematic of the CPA assay with optogenetic activation of the ACC in CFA-treated rats. Light stimulation was paired with PP in one chamber during the conditioning phase, and the other chamber was paired with NP. **b** Expression of YFP-ChR2 in the ACC. From left to right: low magnification (×20) view of ChR2-eYFP in the ACC; high-magnification (×100) view of CaMK II staining, YFP-ChR2 staining, DAPI staining, and Merged images. Arrows point to sample co-stained neurons. **c**, **d** ACC activation blocked the anti-aversive effects of ketamine in CFA-treated rats. **c** CPA test for YFP rats that received CFA and ketamine treatments; *n* = 8; *p* = 0.0381, paired Student’s *t*-test. **d** CPA test for ChR2 rats that received CFA and ketamine treatments; *n* = 9; *p* < 0.0001. **e** The anti-aversive effect of ketamine in CFA rats was eliminated by the activation of ACC, as shown by the increased CPA score in the ChR2 group; *n* = 8–9; *p* = 0.0133, unpaired Student’s *t*-test. **f** Activation of the ACC did not alter locomotion; *n* = 7; *p* = 0.4348, unpaired Student’s *t*-test. Error bars represent S.E.M. Scale bar equals 50 μm in **b**. ^∗^*p* < 0.05; ^∗∗∗∗^*p* < 0.0001

Previous studies have shown that the inhibition of ACC activities can reduce pain aversion^8,27^. To verify that inhibition of ACC hyperactivity is a mechanism for the enduring anti-aversive effect of ketamine in the chronic pain state, we used optogenetics and in vivo pharmacology to alter the activity levels of excitatory pyramidal neurons in the ACC of CFA-treated rats. First, we used a CAMKII (Ca^2+^/calmodulin-dependent protein kinase II) promotor to express channelrhodopsin-2 (ChR2) or control (yellow fluorescent protein (YFP)) vector in the pyramidal neurons of the ACC, and paired light treatment with PP stimulation in one chamber, and no light treatment or noxious stimulation in the opposite chamber, during the conditioning phase of CPA assays (Fig. 6a, b). Conditioning with light activation of the excitatory ACC neurons blocked the anti-aversive effects of ketamine (Fig. 6c, d), as ChR2-expressing rats demonstrated enhanced CPA scores compared with control (YFP) rats (Fig. 6e). These results suggest that inhibition of neuronal activity in the ACC likely mediates the anti-aversive effect of ketamine. Activation of the ACC did not alter baseline locomotion of rats (Fig. 6f).

**Fig. 7:**
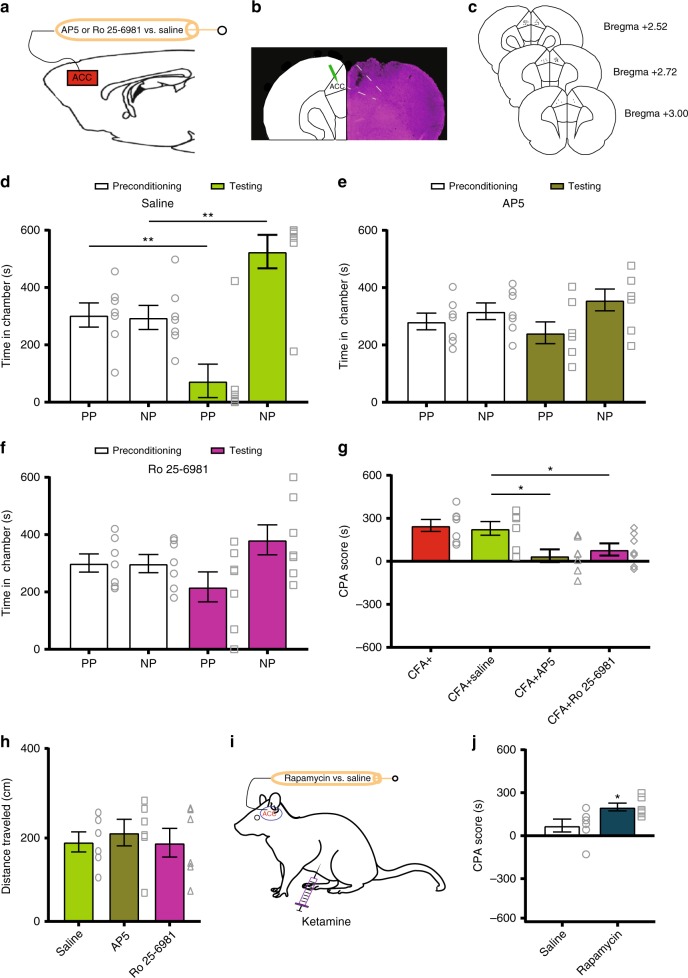
NMDA receptor antagonism in the ACC inhibits pain aversion. **a** Schematic of intra-ACC drug infusions. **b** Representative brain slice showing the injection site in the ACC. **c** Location of intracranial injections. **d** Intra-ACC administration of saline did not alter the aversive response to acute pain in CFA-treated rats; *n* = 7; *p* = 0.0028, paired Student’s *t-*test. **e** CFA-treated rats showed no avoidance of the PP chamber after intra-ACC AP5 injection; *n* = 7; *p* = 0.4035. **f** Intra-ACC administration of an NR2B antagonist (Ro 25–6981) reduced the aversive response to noxious stimulations; *n* = 7; *p* = 0.0957, paired Student’s *t*-test. **g** The inhibition of NMDA receptors in the ACC provided anti-aversive effects in the chronic pain state; *n* = 7, *p* = 0.0122 (CFA+saline vs. CFA+AP5), *p* = 0.0387 (CFA+saline vs. CFA+Ro 25–6981), unpaired Student’s *t*-test. **h** Neither AP5 nor Ro 25–6981 affected locomotion; *n* = 6; *p* = 0.5893 (AP5 vs. saline), *p* = 0.9627 (Ro 25–6981 vs. saline), unpaired Student’s *t-*test. **i**, **j** Pre-treatment of rapamycin in the ACC blocked the anti-aversive effect of ketamine in CFA-treated rats, as demonstrated by an increased CPA score; *n* = 6; *p* = 0.0334, unpaired Student’s *t*-test. Error bars represent S.E.M. ^∗^*p* < 0.05; ^∗∗^*p* < 0.01

Ketamine is known to block NMDA receptors to modify cortical synaptic plasticity^23^. In order to understand if NMDA receptors in ACC neurons mediate the anti-aversive effects of ketamine, we injected AP5, a selective blocker of NMDA receptors, in the ACC of CFA-treated rats (Fig. 7a–c). Intra-ACC injection of AP5 blocked the aversive response to noxious stimulation in the chronic pain state (Fig. 7d, e). To further demonstrate the specificity of the NMDA receptor block, we administered an inhibitor for NR2B receptors, Ro 25-6981. Our results indicate that NR2B antagonism also generated an anti-aversive response in CFA-treated rats (Fig. 7f). The NR2B receptor subunit is known to be important for synaptic plasticity. Previous studies have demonstrated that enhancement of NR2B expression in the ACC contributes to the chronic pain state^43^. Both AP5 and Ro 25–6981 reduced CPA scores (Fig. 7g), without impacting basic locomotion (Fig. 7h). Interestingly, Ro 25–6981 did not block the anti-aversive response to CFA to the same level as ketamine, possibly due to the low dose or involvement of other NMDA receptor subunits. Overall, however, these results strongly suggest that NMDA receptor antagonism in the ACC contributes to the mechanism for ketamine to inhibit altered aversive processing in the chronic pain state.

Previous studies on the effect of ketamine in depression demonstrated that the NMDA receptor blockade could induce the activation of mTORC1 (mammalian target of rapamycin complex 1), a key translational regulator^23,44,45^. Thus, we tested the role of mTORC1 in mediating the anti-aversive effect of ketamine. We delivered rapamycin, a specific blocker of mTORC1, into the ACC, prior to ketamine infusion (Fig.7i). We found that rapamycin blocked the anti-aversive effect of ketamine (Fig. 7j). Thus, mTORC1 is likely involved in the mechanism of ketamine for its enduring relief of the affective component of pain.

## Discussion

A key feature of chronic pain is the amplified affective response to nociceptive inputs in a generalized, anatomically nonspecific manner. In the current study, we have shown that a single sub-anesthetic dose of ketamine can reduce hyperactivity of the neurons in the ACC to specifically inhibit the affective symptoms of chronic pain over a prolonged period of time. Thus, our results demonstrate the feasibility to selectively target the abnormal affective expression of chronic pain as a novel therapeutic approach.

A number of previous studies have demonstrated a heightened aversive response, secondary to nociceptive inputs from the site of chronic pain^19,25,27,46^. Our results here extend beyond these results to show that chronic pain at one site in the body can increase the aversive response to acute mechanical pain from a different location. These results are compatible with two previous studies on the evaluation of aversive responses in rodents to thermal and mechanical noxious stimulations in the chronic pain state^8,30^. These results in animal models confirm the epidemiological findings in clinical pain syndromes, such as fibromyalgia and persistent postoperative pain^2–6^. In these chronic pain syndromes, patients can present with magnified aversive reactions that are not associated with any particular sensory pathways or locations. As the result, therapies that target specific peripheral or spinal sensory pathways may not work, whereas treatments that selectively inhibit the affective processing in the brain can achieve a greater impact.

The ACC is a region that is well known to process and regulate the affective component of pain^11–21^. Individual neurons in the ACC have been shown to respond to noxious stimulation by increasing firing rates^31–35,47,48^, and hyperactivity of the ACC neurons has been observed in a number of animal studies^7,8,49^. Two recent studies particularly highlighted the importance of ACC hyperactivity in the chronic pain state^8,50^. In these studies, neurons in the ACC were found to demonstrate increased firing rates in chronic pain models. Furthermore, optogenetic activation of the ACC neurons enhanced the aversive response to pain, whereas the inhibition of these neurons reduced pain aversion. Our results here show that ketamine has a similar effect as optogenetic inhibition of the ACC. The ACC is part of the medial pain pathway. Structures within the medial pain circuits receive ascending nociceptive inputs bilaterally and project diffusely to a number of cortical and subcortical targets. Unlike the somatosensory cortex, the ACC is not known to possess an anatomically specific somatotopic map. Thus, it is not surprising that in the chronic pain state, synaptic plasticity in the ACC can lead to enhanced aversive processing in response to diffuse noxious inputs. As a result, drugs that inhibit these maladaptive changes have the potential to reset the scale of aversive valuation and response.

In contrast to the ACC, an adjacent area in the rodent cortex, the prelimbic PFC (PL-PFC) is known to provide pain inhibition when activated^26,51^. Interestingly, whereas chronic pain increases the basal and noxious stimulus-induced firing rates in the ACC, it has the opposite effect on the PL-PFC^30^. Thus, a reduction in PFC activities and an enhancement in ACC activities may play potentially complementary roles in the chronic pain state. Indeed, the ACC is only one of the key nodes in a complex network of cortical and subcortical structures that process and regulate pain aversion. Other regions such as the amygdala, insular cortex, periaqueductal gray, and nucleus accumbens may also play important roles in the abnormal aversive response in the chronic pain state, as well as the anti-aversive effects of ketamine.

Ketamine has been used primarily as a general anesthetic or acute analgesic. Its anti-nociceptive effect is short-lived, and hence it is ideally suited for acute pain management. The use of ketamine in chronic pain syndromes has mixed results, and it generally involves prolonged, continuous infusions that are given repeatedly over days to weeks^52–57^. On the other hand, ketamine has been shown to be highly effective as an antidepressant, as a single dose can produce up to 1 week of symptom relief^58,59^. Animal studies have revealed several potential mechanisms for this antidepressant effect. For example, inhibition of the NMDA receptors can lead to downstream increases in brain derived neurotrophic factor (BDNF) expression in the hippocampus and PFC, relevant regions for affect regulation^24,60^. In addition, ketamine can promote the expression of specific synaptic and intracellular signaling proteins in the cortex, through the upregulation of mTORC1, a translational regulator^23,44,45^. Our finding that ketamine can produce enduring suppression of ACC activities through NMDA receptor antagonism provides an important mechanism for this analgesic in brain circuits. Furthermore, in our study, mTORC1, which has been shown to mediate the antidepressant property of ketamine, appears to facilitate its anti-aversive effects as well. mTORC1 is a key regulator of protein translation, and additional studies are needed to investigate which proteins are specifically targeted in the ACC by the activation of mTORC1 through ketamine, in the context of chronic pain. Future studies are also needed to examine additional molecular and synaptic mechanisms for the anti-aversive effects of ketamine in the ACC as well as other brain regions. Meanwhile, the temporal parallel between the antidepressant effect and anti-aversive effect of ketamine suggests that the mechanism proposed in the current study may be applicable in other neuropsychiatric conditions. In contrast, the temporal dissociation between the anti-nociceptive and anti-aversive effects of ketamine indicates that this drug likely has multiple molecular and circuit targets to mediate different therapeutic functions. It also highlights the ability for ketamine to separate the affective from the sensory symptoms of chronic pain.

Whereas we recorded from layers 5–6 of ACC, a limitation of our technique is that we could not be absolutely certain about the identity of excitatory and inhibitory neurons. Nevertheless, when we sorted these neurons using conventional methods^61^, we found that most of the neurons activated by ketamine were pyramidal neurons, compatible with previous studies using in vivo electrophysiology to investigate cortical neurons^39–41^. Thus, the effects that ketamine had on spike rates are likely mediated through pyramidal neurons. In addition, in our optogenetic studies, we used a promotor that is specific for excitatory neurons. By showing that their activation blocked the effect of ketamine, we provided additional support for the role of these neurons in the ACC in the anti-aversive effects of ketamine. However, future studies are still needed to definitively investigate the impact of ketamine on interneurons in the ACC in the chronic pain state, and techniques such as juxtacellular labeling may be helpful in such studies^62–65^.

In conclusion, we have found that a single sub-anesthetic dose of ketamine can reduce ACC hyperactivity to specifically inhibit the affective symptoms of chronic pain over a prolonged period of time. These results demonstrate that the affective component of pain can be dissociated from the sensory components, and selective inhibition of the affective state can have a profound impact on the treatment of chronic pain.

## Methods

### Experimental animals

Male Sprague-Dawley rats were purchased from Taconic Farms (Albany, NY) and kept at Mispro Biotech Services Facility in the Alexandria Center for Life Science, with controlled humidity, temperature, and 12 h (6:30 AM to 6:30 PM) light–dark cycle. Food and water were available ad libitum. Animals arrived to the animal facility at 250 to 300 g and were given on average 14 days to adjust to the new environment prior to the onset of experiments.

### Drugs

To establish the chronic inflammatory pain model in rats, 0.06 mL of CFA (*Mycobacterium tuberculosis*, Sigma-Aldrich) was suspended in an oil–saline (1:1) emulsion and injected subcutaneously into the plantar aspect of the hind paw opposite to the paw that was stimulated by pin prick. Control groups received an equal volume of saline injections. Ketamine hydrochloride (Ketaset) was purchased from Zoetis. Rats received 5, 10, or 20 mg kg^−1^ ketamine injection intraperitoneally in the ketamine group (0.5 mL), whereas a similar volume of saline was injected intraperitoneally to the control group. D-(-)-2-Amino-5-phosphonopentanoic acid (AP5, 25 mM, Abcam) and the NR2B subunit antagonists Ro 25-6981 (2 μg μL^−1^, Sigma-Aldrich) were diluted in sterile normal saline, and 0.5 μL was injected in bilateral ACC. Control group received an equal volume of saline. Rapamycin (10 nmol per 0.5 μL, Sigma-Aldrich) or a vehicle was delivered into the ACC approximately 30 min prior to intraperitoneal ketamine infusions.

### Spare nerve injury surgery

After rats were anesthetized with isoflurane (1.5 to 2%), the skin on the lateral surface of the left thigh of the rat was incised^29,51,66^. A section was subsequently made through the biceps femoris muscle to expose the sciatic nerve and its three terminal branches: sural, common peroneal, and tibial nerves. The common peroneal and tibial nerves were tied with nonabsorbent 5-0 silk sutures at the proximal point of trifurcation. The nerves were then cut distal to each knot, and approximately 5 mm of the distal ends were removed to prevent reattachments. Nerves were dissected but not cut in sham surgeries (control group). The muscle layer was then sutured closed, and the skin was stapled. Staples were removed before any behavioral experiments.

### Virus construction and packaging

Recombinant AAV vectors were serotyped with AAV1 coat proteins and packaged at the UPenn vector core. Viral titers were 5 × 10^12^ particles per mL for AAV1.CaMKII.ChR2-eYFP.WPRE.hGH, and AAV1.CaMKII(1.3).eYFP.WPRE.hGH.

### Stereotaxic optic fiber implantation and viral injection

Rats were anesthetized with isoflurane (1.5 to 2%). In all experiments, virus was delivered to the ACC only. Rats were bilaterally injected with 0.5 µL of viral vectors at a rate of 0.1 µL every 10 s with a 26-gauge 1 µL Hamilton syringe at anteroposterior (AP) +2.6 mm, mediolateral (ML) ±1.6 mm, and dorsoventral (DV) −2.25 mm, with tips angled 28° toward the midline. Rats were then implanted with 200 μm optic fibers held in 1.25 mm ferrules (Thorlabs) in the ACC: AP +2.6 mm, ML ±1.6 mm and DV −1.75 mm. Fibers with ferrules were held in place by dental acrylic^8^.

### Cannula implantation and intracranial injection

For cannula implantation^51^, rats were anesthetized with isoflurane (1.5 to 2%). Rats were stereotaxically implanted with two 26-gauge guide cannulas (PlasticsOne, USA) bilaterally in the ACC with coordinates as follows: 2.6 mm anterior to bregma; 1.6 mm lateral to the sagittal suture, tips angled 28° toward the midline, and 1.25 mm ventral to skull surface. Cannulas were held in place by dental acrylic. For intracranial injections, solutions were loaded into two 30 cm lengths of PE-50 tubing attached at one end to 10 μL Hamilton syringes filled with distilled water and at the other end to a 33-gauge injector, which extended 1.0 mm beyond the implanted guides for the ACC. Over a period of 100 s, 0.5 μL solution was injected slowly into the ACC bilaterally. Injector cannulas were kept in place for 60 s before removal from guides to ensure diffusion of solution. Stylets were replaced after the removal of injector cannulas from cannula guides, and animals were subject to behavior tests.

### Electrode implant and surgery

Stereotrodes were constructed from two twisted 12.7 µm polyimide-coated microwires (Sandvik) and mounted in a VersaDrive8 (Neuralynx)^8,67^. Electrode tips were plated with gold to reduce electrode impedances to 100–500 kΩ. During implantation, rats were anesthetized with isoflurane (1.5–2%). The skull was exposed and a craniotomy was performed over unilateral anterior cingulate cortex (AP +2.5–3.5 mm, ML 0.8–1.8 mm). The electrode bundle was lowered slowly at DV 1.6 mm with the tip angled 10° toward the midline. The drive was secured to the skull screws with dental cement. Rats were given on average 1 week to recover before neural recordings.

### In vivo electrophysiological recordings

Animals with chronic electrode implants were given a 30 min period to habituate to a recording chamber over a mesh table before stimulation^8^. Noxious stimulation by pricking with a 27-gauge needle (PP) was applied to the plantar surface of the hind paw contralateral to the brain recording site in free-moving rats. Noxious stimulation was terminated by paw withdrawals. All recording sessions consisted of approximately 30 trials with variable inter-trial intervals (approximately 1 min). A 120 fps video camera (DMK23U, image source) was used to record the experiment. Long inter-trial intervals between trials were used to avoid sensitization. No behavioral sensitization or physical damage to the paw was observed.

### Neural data collection and preprocessing

Stereotrodes were lowered in steps of 60 µm before each day of recording. The neuronal activity and the onset of pin prick stimulation were simultaneously recorded with acquisition equipment (OmniPlex D with Digital Headstage Processor, Plexon). Signals were monitored and recorded at a sample rate of 40 kHz. To get spike activity, the raw data were band pass filtered from 300 Hz to 7.5 kHz with subsequent offline sorting, using commercial software (Offline Sorter, Plexon). Trials were aligned to the initiation of the peripheral stimulus to compute the peri-stimulus time histograms (PSTHs) for each single unit using MATLAB (Mathworks).

### Immunohistochemistry

Rats were deeply anesthetized with isoflurane and transcardially perfused with ice-cold phosphate-buffered saline (PBS) and paraformaldehyde (PFA). Brains were fixed in PFA overnight and then transferred to 30% sucrose in PBS to equilibrate for 3 days^51^. Then, 20 µm coronal sections were collected using Microm HM525 Cryostat (Thermo Fisher Scientific), washed in PBS, and coverslipped with Vectashield mounting medium. Images containing electrodes or cannula were stained with cresyl violet or hematoxylin and eosin stain, and viewed and recorded under a Nikon eclipse 80i microscope with a DS-U2 camera head. Sections were also made after viral transfer for opsin verification, and these sections were stained with anti-rabbit GFP (1:500, Abcam, Cambridge, MA, #AB290), CaMKII-α (6G9) mouse monoclonal antibody (1:100, Cell Signaling Technology, Danvers, MA, USA #50049), and 4′,6-diamidino-2-phenylindole (DAPI; 1:200, Vector Laboratories, Burlingame, CA) antibodies. Secondary antibodies were anti-rabbit IgG conjugated to AlexaFluor 488, and anti-mouse IgG conjugated to AlexaFluor 647 (1:500, Life Technologies, Carlsbad, CA). Images were acquired with a Zeiss LSM 700 Confocal Microscope (Carl Zeiss, Thornwood, NY). Animals with improper fiber or electrode or cannula placements, low viral expression, or viral expression outside the ACC were excluded from further analysis.

### Animal behavioral tests

For optogenetic experiments, optic fibers were connected to a 473 nm (for ChR2) laser diode (Shanghai Dream Lasers) through a mating sleeve as shown in previous studies^51^. Laser intensity was calibrated with a power meter (Thorlabs) prior to experiments. Laser was delivered using a TTL pulse-generator (Doric).

### Conditioned place aversion

CPA experiments were conducted in a two-chamber device^8,26^. Animal movements in each chamber were recorded by a high-speed camera and analyzed with the AnyMaze software. The CPA protocol included preconditioning (baseline), conditioning, and testing phases (10 min during each phase). The two chambers were connected. Animals spending more than 500 s or less than 100 s of the total time in either chamber in the preconditioning phase were eliminated from further analysis. Immediately following the preconditioning phase, the rats underwent conditioning for 10 min. During conditioning, one of the two chambers was paired with a PP. The PP stimulus was repeated every 10 s. During the optogenetic experiments, light activation was concurrent with noxious stimulation in one of the treatment chambers. PP, optogenetic stimulation, and chamber pairings were counterbalanced. During the test phase, the animals did not receive any treatment and had free access to both compartments for a total of 10 min. Animal movements in each of the chambers were recorded, and the time spent in either of the treatment chambers was analyzed by the AnyMaze software. Decreased time spent in a chamber during the test phase as compared with the baseline indicates avoidance (aversion) for that chamber.

### Mechanical allodynia test

A Dixon up-down method with von Frey (VF) filaments was used to measure mechanical allodynia^51^. Before tests, rats were individually placed into plexiglass chambers over a mesh table and acclimated for 20 min. Beginning with 2.55 g, VF filaments in a set with logarithmically incremental stiffness (0.45, 0.75, 1.20, 2.55, 4.40, 6.10, 10.50, 15.10 g) were applied to the paws of rats. The 50% withdrawal thresholds were calculated using an up-down method^51^.

### Locomotion

We recorded locomotion over 5 min for rats that received optogenetic activation or intracranial injection. During light activation, blue light was turned on for 3 s every 10 s (to mimic the conditioning phase of the CPA assays) during the locomotion test. Total distance traveled was computed based on AnyMaze recordings.

### Statistical analysis

The results of behavioral experiments were given as mean ± S.E.M. For mechanical allodynia, a two-way analysis of variance (ANOVA) with repeated measures and post-hoc multiple pair-wise comparison Bonferroni tests or unpaired *t*-tests were used whenever appropriate. For the CPA tests, paired Student’s *t*-test was used to compare the time spent in each treatment chamber before and after conditioning (i.e., baseline vs test phase for each chamber). Decreased time spent in a chamber during the test phase as compared with the baseline indicates avoidance (aversion) for that chamber. A CPA score was computed by subtracting the time spent in the noxious (PP) chamber during the test phase from the time spent in that chamber at baseline^8,26^. A two-tailed unpaired Student’s *t*-test was used to compare differences in CPA scores under various testing conditions.

For neuronal spike analysis, we calculated PSTHs using a 5 s range before and after peripheral stimulus^30^. The number of spikes in each stimulus-aligned bin was averaged across all trials to create the PSTH. We then calculated basal spontaneous firing rates for each neuron to be the average of the PSTH bins before the onset of PP stimulation, and peak pain-evoked firing rates to be the maximum value of the PSTH after stimulus onset (within 5 s from the stimulus). Neuronal firing rates had a non-Gaussian distribution, compatible with previous reports^39^. Thus, nonparametric tests were performed. To compare firing rates of rats before CFA injection with rats after CFA injection and either saline or ketamine administrations, we used Kruskal–Wallis test with post-hoc Dunn’s multiple comparisons.

For all tests, a *p* value < 0.05 was considered statistically significant. All data were analyzed using the GraphPad Prism Version 7 software (GraphPad) and MATLAB (MathWorks).

## Electronic supplementary material

Supplementary Information

## Data Availability

The authors declare that all the data supporting the findings of this study are available within the paper and its supplementary information files, or will be made available upon reasonable request.
