# Supplementary material for: Ketamine reduces aversion in rodent pain models by suppressing hyperactivity of the anterior cingulate cortex
Source: Nat Commun. 2018 Sep 14;9:3751. doi: 10.1038/s41467-018-06295-x (PMC6138720; doi:10.1038/s41467-018-06295-x)
Supplement: Supplementary file 1 — Supplementary Information [file 41467_2018_6295_MOESM1_ESM.pdf]

Supplementary Information

Titile: Ketamine Reduces Aversion in Rodent Pain Models by Suppressing Hyperactivity of the Anterior Cingulate Cortex

Zhou et al.

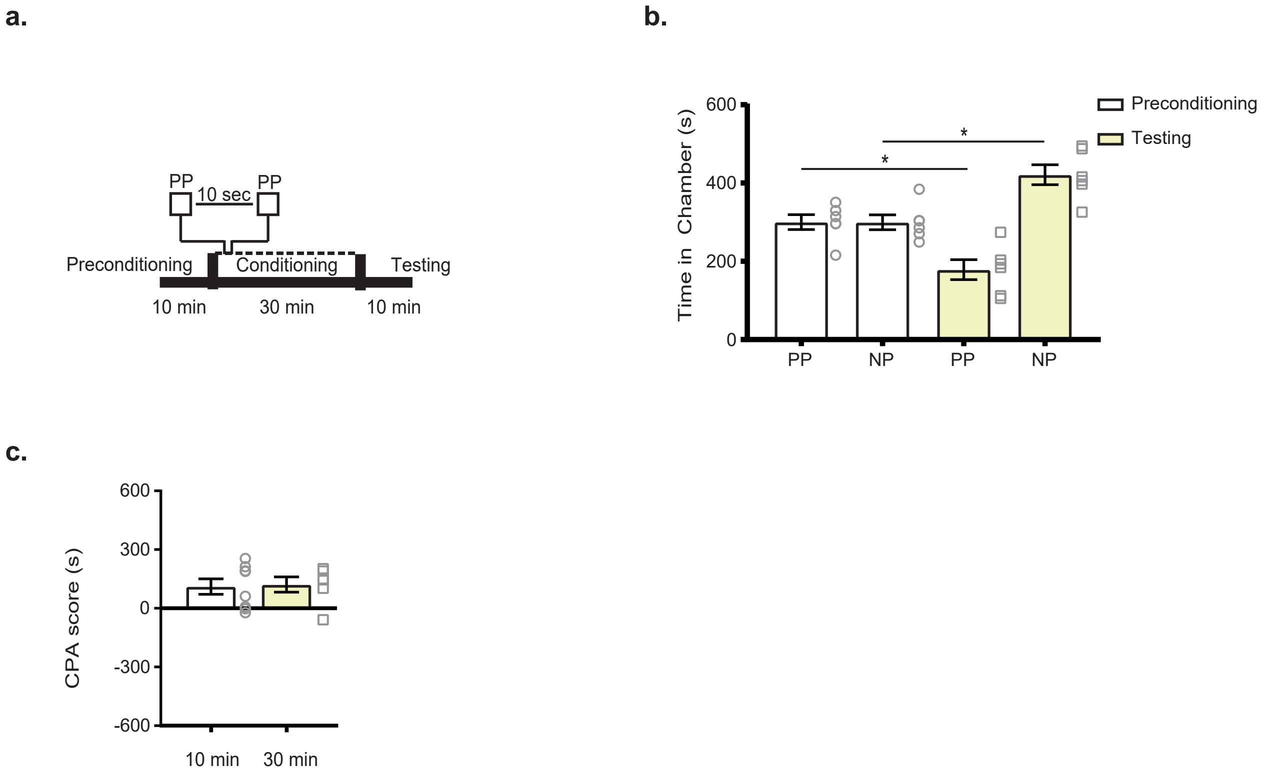

Supplementary Fig. 1. An extended duration of the conditioning phase did not alter the aversive response to acute pain. a Protocol of a 30-min conditioning phase for the CPA assay. **b** After 30 minutes of conditioning, naïve rats displayed avoidance to the chamber paired with a noxious stimulus (PP). n=6; p=0.0261, paired Student's t test. **c** The 30-min conditioning protocol did not alter the aversive response to acute noxious stimulations, compared with the 10-min conditioning protocol. n=6-7; p=0.8577, unpaired Student's t test. Error bars represent S.E.M.

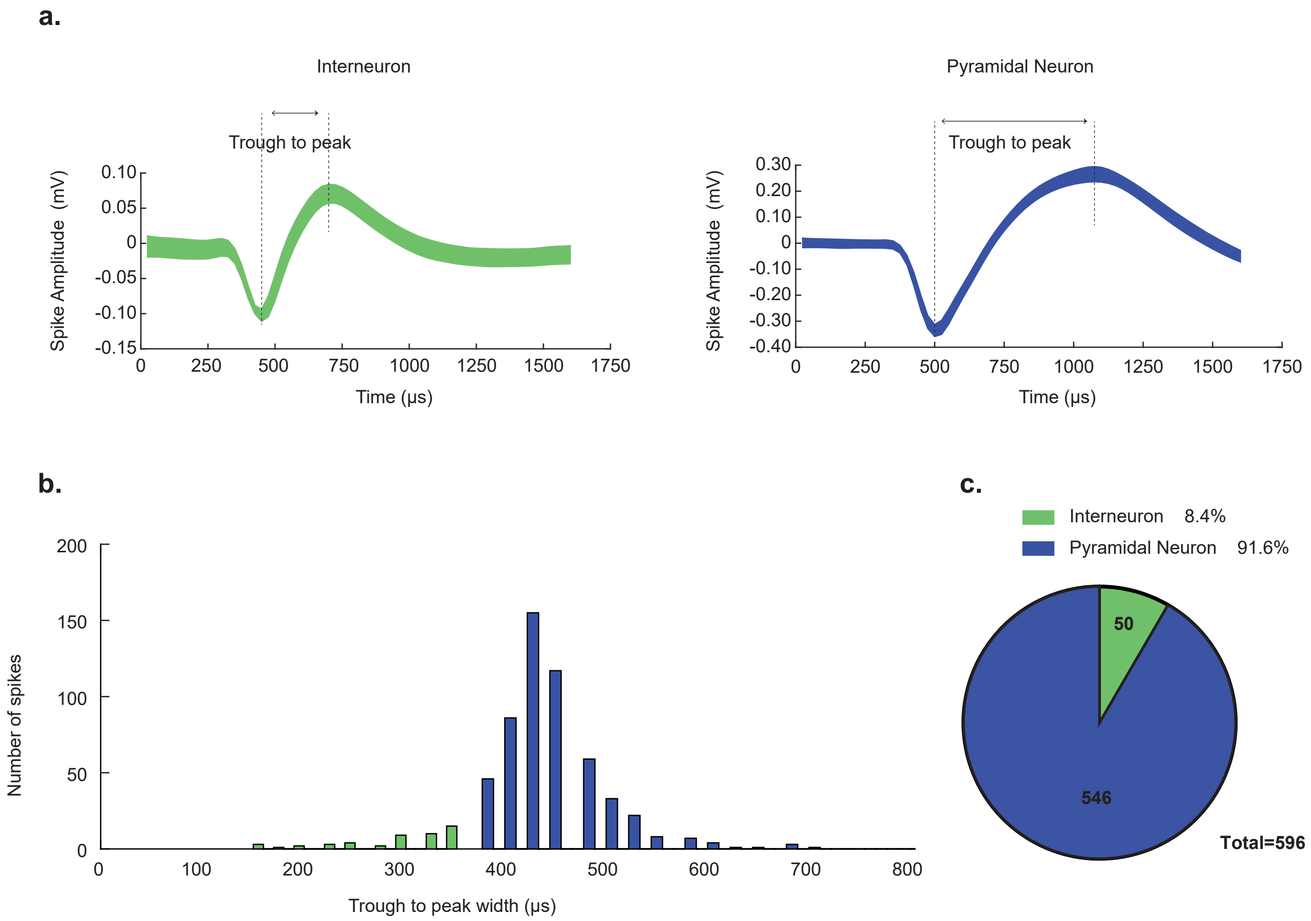

Supplementary Fig. 2. Identification of recorded neurons in the ACC. a Example spike waveforms of an interneuron and a pyramidal neuron**. b** Histogram of spike trough to peak widths. Interneurons have shorter trough to peak widths (<360 μs), while pyramidal neurons have longer widths. **c** Proportions of recorded interneurons and pyramidal neurons in the ACC.
